# Comparison of biomechanical performance of single-level triangular and quadrilateral profile anterior cervical plates

**DOI:** 10.1371/journal.pone.0250270

**Published:** 2021-04-15

**Authors:** Fu Cao, Rongchang Fu, Wenyuan Wang

**Affiliations:** School of Mechanical Engineering, Xinjiang University, Urumqi, China; University of California San Francisco, UNITED STATES

## Abstract

The quadrilateral anterior cervical plate (ACP) is used extensively in anterior cervical discectomy and fusion (ACDF) to reconstruct the stability of the cervical spine and prevent cage subsidence. However, there have been no comparison studies on the biomechanical performance of quadrilateral ACP and triangular ACP. The objective of this study is to investigate the functional outcomes of quadrilateral ACP and triangular ACP usage in ACDF surgery. In this study, a finite element model of intact C1-C7 segments was established and verified. Additionally, two implant systems were built; one using triangle anterior cervical plates (TACP) and another using quadrilateral orion anterior cervical plate (QACP). Both models were then compared in terms of their postoperative biomechanical performance, under normal and excessive motion. Compared to QACP, the peak stress of the TACP screws and plates occurred at 359.2 MPa and 97.2 MPa respectively and were the highest during over extension exercises. Alternately, compared to TACP, the endplate peak stress and the cage displacement of QACP were the largest at over extension, with values of 7.5 MPa and 1.2 mm, respectively. Finally, the average stress ratio of bone grafts in TACP was relatively high at 31.6%. In terms of biomechanical performance, TACP can share the load more flexibly and reduce the risks of cage subsidence and slippage but the screws have high peak stress value, thereby increasing the risk of screw slippage and fracture. This disadvantage must be considered when designing a TACP based implant for a potential patient.

## Introduction

Since the 1980s, anterior cervical discectomy and fusion (ACDF), using a bone graft-intervertebral cage, was the gold standard in the treatment of cervical spondylotic radiculopathy, myelopathy, trauma, and other cervical spondylopathy [1–3]. However, some studies have reported cage slippage and subsidence in 40% of the patients, after implantation, causing delayed or no bone graft fusion [3, 4]. To prevent subsidence or displacement during a fusion process and to promote an increased fusion rate, an additional anterior cervical plate (ACP) system must be designed to enhance cage stability and avoid fusion failure [5]. In fact, the ACP addition to the ACDF procedure, due to its great clinical benefit, is the most preferred and widely used in surgeries today. However, the ACP included ACDF surgeries are not without complications. Multiple reports have demonstrated loose screws and fracture, cervical plate fracture and displacement, cage subsidence, and bone graft nonunion caused by stress shielding [5, 6]. As a result, no general consensus has been reached on the ideal device for increasing fusion rate while decreasing complications due to surgery or healing. Several studies have attempted to address this issue. Şimşek et al. [7], for example, designed a hinged quadrilateral cervical plate to reduce the potential of screw loosening and fracture due to bending of the spine. Peterson et al. [8], on the other hand, examined the relationship between cervical plate stiffness and fusion using quadrilateral cervical plate of varying stiffness. Conversely, Mackiewicz et al. [9] explored the differences in the stability of different types of plates after a single-segment ACDF surgery; namely static trapezoidal Casper cervical plate, quadrilateral Casper cervical plate, and dynamic cervical plate. ACP used in single-level ACDF surgery, are mostly designed to be rectangular or rectangle-like quadrilaterals. Moreover, in a single level ACDF surgery, a quadrilateral cervical plate needs to be equipped with at least four screws for fixation and four threaded holes for proper fixation. In a triangular cervical plate, however, only three screws and three threaded holes can suffice. This allows the triangular cervical plate to produce shorter operation time, reduce screw damage to the vertebrae, and so on. Given the benefits of a triangular cervical plate over a quadrilateral cervical plate, it is imperative to perform a comprehensive comparison analysis between the two ACP systems. In order to evaluate the triangle fixation system biomechanics performance, this study designed the triangle anterior cervical plate (TACP) and quadrilateral Orion anterior cervical plate (QACP) to compare the surgical outcomes of both ACP systems in terms of slippage and fracture of the plates and screws, the slippage and subsidence of the cage, and the stress ratio of the bone grafts.

## Materials and methods

### The establishment of an intact cervical spine C1-C7 model

A digital model establishment of animal cervical vertebrae was used to analyze the finite element (FE) method. This allowed data, that was difficult to obtain clinically and experimentally, to be displayed using FE calculation results. Moreover, the established cervical spine model was able to reproduce injuries and clinical operations, which enabled us to successfully study the prevention, diagnosis, and treatment of spinal diseases [10–12]. In this study, a 24-year-old, 65 kg weight, 181 cm height healthy adult cervical spine computed tomography (CT) scan was performed (This work was approved by the Ethics Committee of the School of Mechanical Engineering of Xinjiang University, and the subject has signed the relevant informed consent). The cervical data was obtained using a CT scan with a DICOM format imported into Mimics (Materialise Inc., Leuven, Belgium). The geometric 3D model of each vertebra was then reconstructed and a spatial geometric configuration of the entire cervical spine was reproduced based on a restored 3D model. Using 3-Matic (Materialise Inc) in the Mimics, a solid model of the C1-C7 vertebrae was created and the intervertebral disc, the facet joint cartilage area, and the starting and ending positions of each ligament were designed. The meshing and material definition of the intact cervical spine solid model were performed in Hyermesh (Altair Engineering, Inc., Troy, Michigan, USA). Our reconstructed vertebral body consisted of the cortical bone, cancellous bone, endplate, and posterior structure. The cortical bone and endplate were defined as thin shells with a thickness of 1 mm. The cortical bone, endplate, and posterior structures were constructed with linear elastic isotropic materials, and the cancellous bone was simulated using the linear elastic anisotropic materials [13]. The intervertebral disc included the matrix, the nucleus pulposus, and the crescentic annulus fibrosus. The nucleus pulposus accounted for about 30%-40% of the entire intervertebral disc volume, consisting of incompressible superelastic materials [14]. Simulating previous studies, the crescentic annulus fibrosus was reconstructed using non-linear elastic isotropic materials [15]. The facet joint cartilage was fashioned with linear elastic isotropic materials and a nonlinear surface-surface contact simulation was performed with a friction coefficient of 0.05 between the cartilage [16]. The ligament section included the anterior longitudinal ligament, posterior longitudinal ligament, ligamentum flavum, interspinous ligament, joint capsular ligament, and posterior capsular ligament and were constructed with non-linear elastic isotropic materials [17], and the ligament positions were determined according to anatomy.

### Fusion system design and modeling

Multiple studies have reported a high incidence of cervical spondylosis in the C5-C6 segment. Therefore, a simulation of cage and anterior cervical plate implantation in the C5-C6 segment was performed [18, 19]. In this study, a restricted QACP, which is commonly used in single-level ACDF surgery was selected for comparison. The size and material of the newly designed TACP met the following requirements: (1) its thickness was consistent with the thickness of the QACP; (2) its height was 5 mm apart from the adjacent intervertebral disc to prevent bone impact between the cervical plate and the anterior edges of the adjacent vertebral body [20]; and (3) its material (medical titanium alloys) was consistent with the material used in QACP.

Medical titanium alloys were selected as the screw material with a length of 14 mm and a diameter of 3.5 mm. In the modeling process, a simplified model without threads was used. Several studies have demonstrated that in a single-level ACDF surgery, screws placed with 32 degrees of scattering outside the sagittal plane and 6 degrees of cohesion in the cross section can maintain a better stability of the fusion system [20, 21]. Therefore, this design was selected for our study.

The conventional cage was built with suitably trimmed polyetheretherketone (PEEK) that was inserted into the C5-C6 intervertebral space. Meanwhile, the cage was designed to maintain a distance of 1.5 mm and 3 mm from the anterior and posterior edges of the vertebral body, respectively, to minimize damages from cage slipping and protrusion into the spinal cord.

Using the above details, two anterior cervical plates were designed, assembled, and used in the simulation of surgical implantation using SolidWork2013 (SolidWorks Corp., MA, USA). In Hypermesh, screws, cervical plates, cages, and bone grafts were constructed using linear elastic isotropic materials.

**Fig 1:**
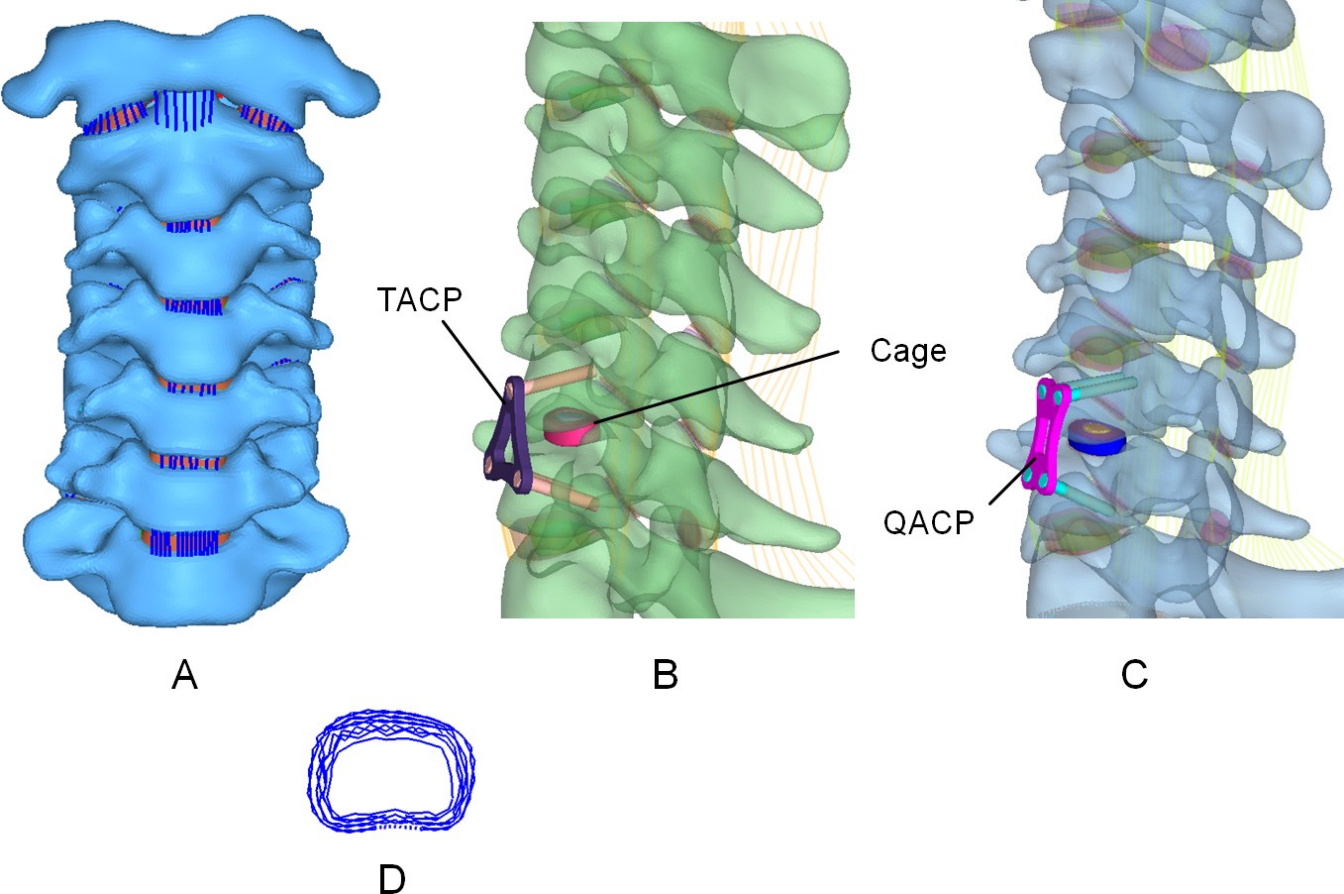
Intact cervical spine C1-C7 model and post-implantation model. (A) The intact C1-C7 cervical spine finite element model; (B) The model after TACP implantation; (C) The model after QACP implantation; (D) crescentic fibrous ring.

The established models are shown in Fig 1. The intact cervical spine model, quadrilateral, and triangular fusion system models were imported into ANSYS 17.0 software (ANSYS, Ltd., USA). The material properties and unit selection of each component were then assigned based on literature review, using the detailed parameters shown in Table 1 [13–17].

**Table 1:** Element type selection and material parameters of the cervical spine model.

Component	Element type	Material properties	Cross-sectional area(*mm*^2^)
Cortical bone	SHELL181	E = 10,000 MPa	
		v = 0.3	
Cancellous bone	SOLID185	E_xx_ = E_yy_ = 100 MPa	
		Ezz = 300 MPa	
		G_yz_ = G_zx_ = 77 MPa	
		v_yx_ = v_zy_ = 0.3	
		v_zx_ = 0.1	
		G_xy_ = 0.3	
Posterior structure	SOLID185	E = 3,500 MPa	
		v = 0.29	
Endplate	SHELL181	E = 450 MPa	
		v = 0.4	
Annulus grounds	SOLID185	E = 3.4 MPa	
		v = 0.4	
Nucleus pulposus	SOLID185	C_10_ = 0.12	
		C_01_ = 0.09	
Annulus fibers	LINK180	E = 175 MPa	
		v = 0.3	
Facet cartilage	SOLID185	E = 10.4 MPa	
		v = 0.4	
Anterior longitudinal	LINK180	E = 30 MPa	6.1
		v = 0.4	
Posterior longitudinal	LINK180	E = 20 MPa	5.4
		v = 0.4	
Ligamentum flavum	LINK180	E = 10 MPa	50.1
		v = 0.4	
Interspinous	LINK180	E = 10 MPa	13.1
		v = 0.4	
Joint capsular	LINK180	E = 20 MPa	50.1
		v = 0.4	
Posterior capsular	LINK180	E = 20 MPa	5
		v = 0.4	
Screw	SOLID185	E = 110 GPa	
		v = 0.34	
Plate	SOLID185	E = 110 GPa	
		v = 0.34	
Cage	SOLID185	E = 3,600 MPa	
		v = 0.31	
Graft bone	SOLID185	E = 3,500 MPa	
		v = 0.3	

### Boundary and loading condition

In the intact C1-C7 models, the C1 vertebral body centroid was calculated and the centroid point was coupled with the C1 vertebral body to prevent stress concentration caused by the concentrated force load. A vertical downward 50N was applied representing the head mass and a 1.0Nm moment was applied in different directions mimicking the flexion, extension, lateral bending, and axial rotation motion of the cervical spine [22]. The constraints of the above three FE models restrict all degrees of freedom of the lower surface of C7. Finally, the model validity was established by analyzing each segment of the model and its range of motions (ROM) under different motion conditions, along with a comparison with ROM in previous literature.

Typically, a patient’s cervical spine ROM should reduce by 46%-89.5% upon ACDF surgery [9]. The moment of 1 Nm before surgery was used to simulate excessive activity in the ACP implant model. The Panjabi et al. [22] study, which was used as a comparison to our models, used a moment of 0.3 Nm to simulate normal motions of its ACP implant models.

## Results

### Model validation

**Fig 2:**
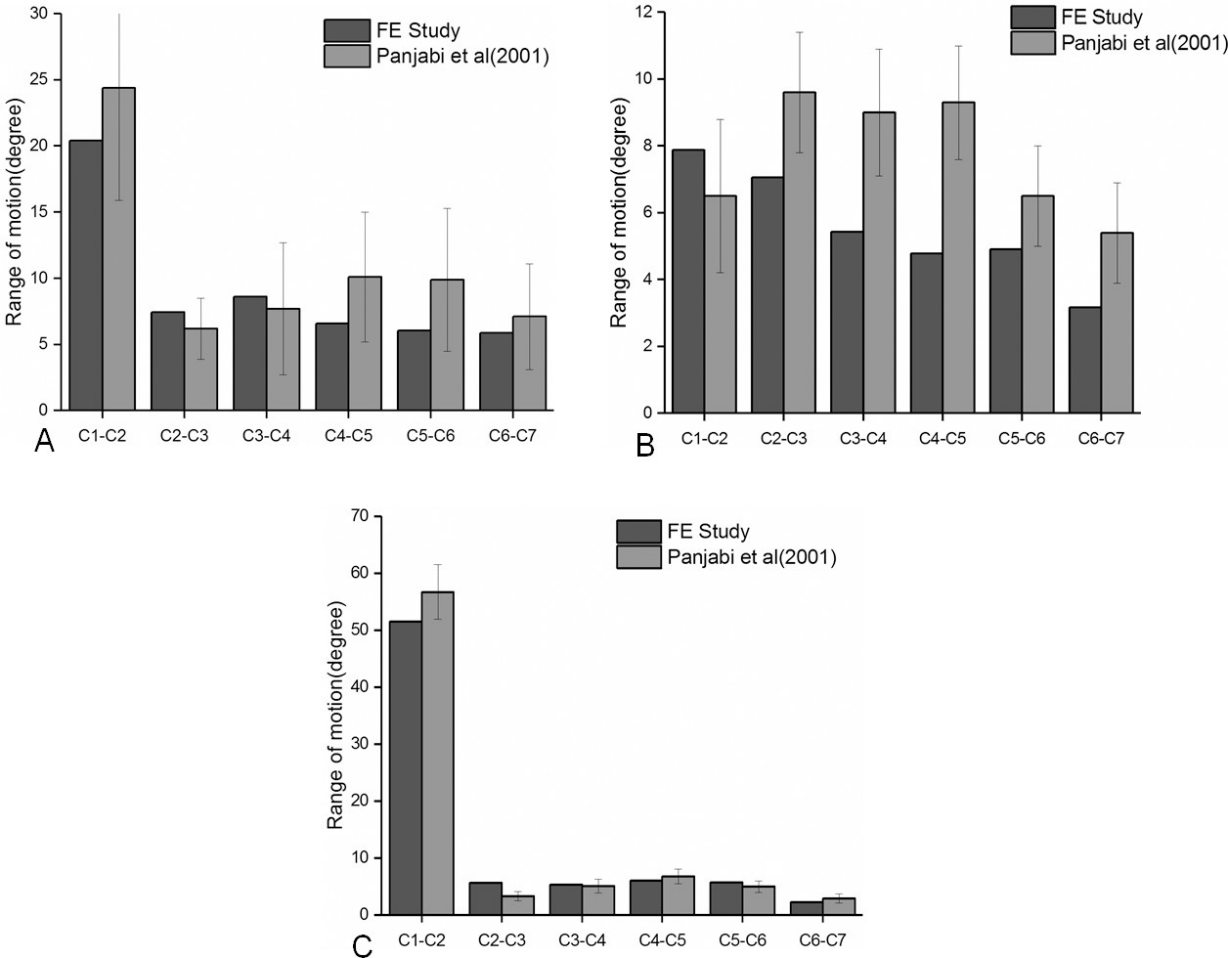
A comparison of the cervical spine ROM analysis under different motion patterns between our study and the vitro experiments. (A) ROM during flexion and extension; (B) ROM during lateral bending; (C) ROM during axial rotation.

This study compared the ROM of C2-C3, C3-C4, C4-C5, C5-C6, and C6-C7 under the motions of flexion, lateral bending, axial rotation, and extension, as reported in previous literature (Fig 2). In the work done by Panjabi et al. [22], all modes of motions generated expected results in terms of ROM movement, expect for the LB motion mode, which generated a large deviation in data. The large deviation in the LB motion mode was also observed in the whole cervical spine model with skull [23]. In Yu et al. [24], the ROM analysis of the C3-C7 segment finite element model showed some discrepancies in the axial motion. There are several possible reasons for these discrepancies. Firstly, differences can be introduced when using experimental data from cadaver spines for FE analysis; Secondly, various research segments may cause diffing in the ROM response; Thirdly, differences in the element type, mesh quality and boundary conditions set by the researcher. And fourth, difference in the profile characteristics of individual cervical spines. As a result, based on these factors, the FE model, established in this study, remained authentic and could generated reliable data for the FE analysis, after simulated TACP and QACP implantation.

### Screw-plate stress

**Fig 3:**
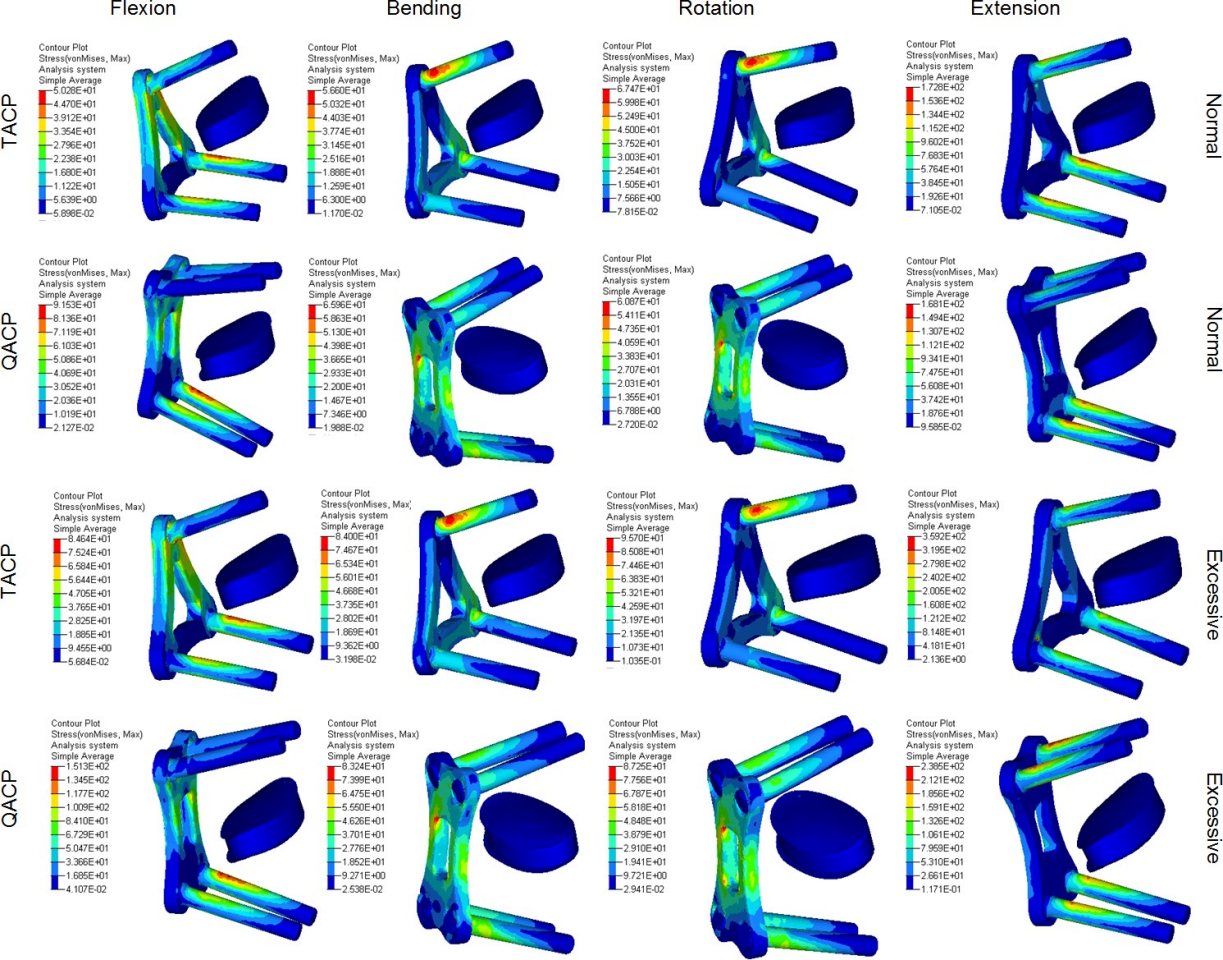
Von-mises equivalent stress cloud diagram examining differences in TACP and QACP surgical outcomes under varying motion conditions. Normal refers to normal postoperative motion, and Excessive refers to excessive postoperative motion.

As shown in the stress cloud diagrams in Fig 3, the stresses on the TACP and QACP screws were concentrated on the root, which was located on the outer edge and therefore was in contact with the cancellous bone. Due to the high stress in this region, this area was prone to screw slippage and fracture. The stresses of the TACP cervical plates, under different motion modes, concentrated pressure on different areas of the cervical plate, thereby distributing the pressure and reducing fracture risk. However, QACP always remained close to the same area, which increased pressure on a localized region and increased fracture risk.

**Fig 4:**
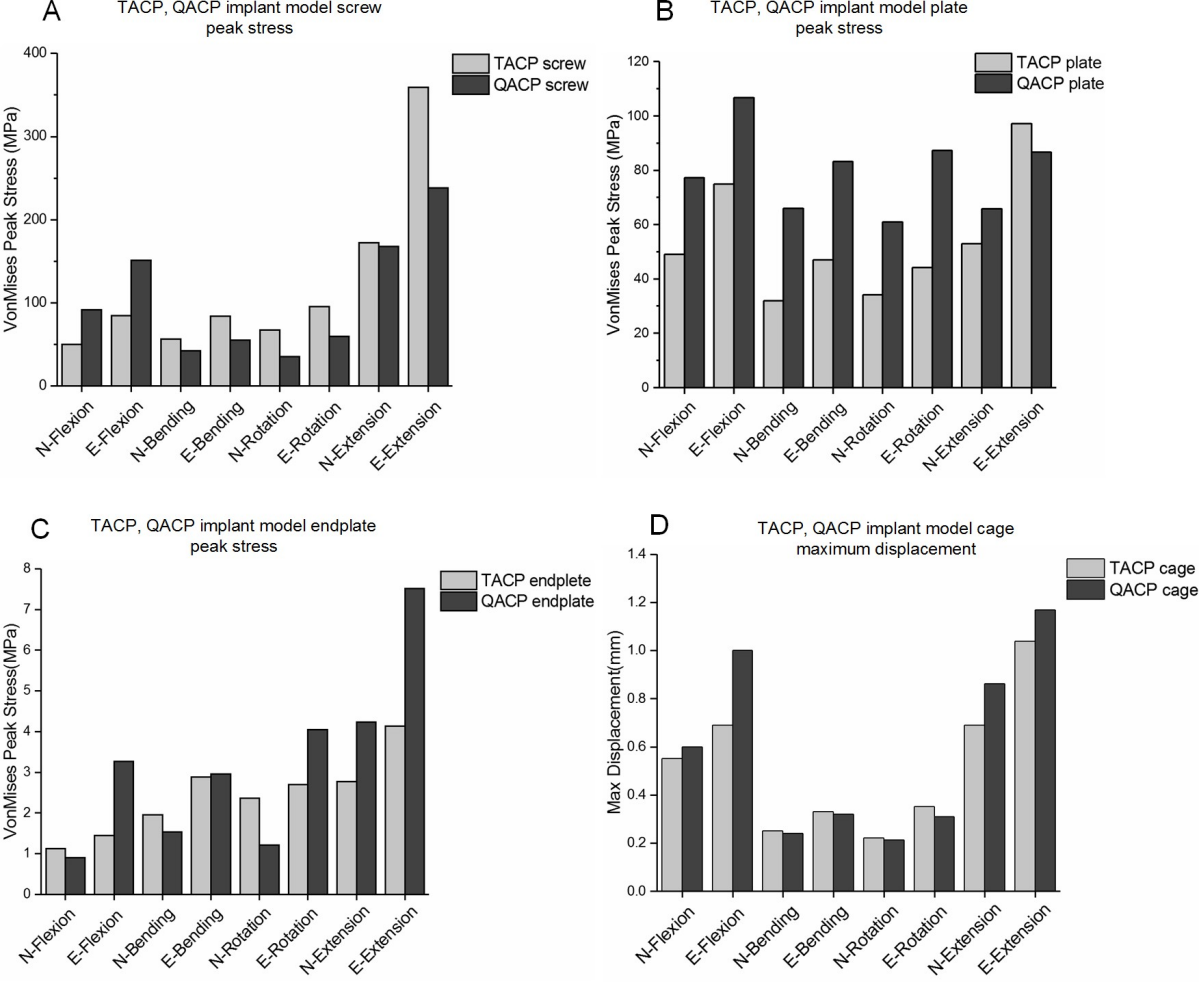
Comparison of the peak stresses in screws, plates, and endplates between triangle anterior cervical plate (TACP) and quadrilateral anterior cervical plate (QACP) systems undergoing normal and excessive motion, and the maximum cage displacement. N, normal postoperative motion; E, excessive postoperative motion. (A) TACP and QACP screw. (B) TACP and QACP cervical plate. (C) TACP and QACP C5-C6 endplate. (D) TACP and QACP cage.

As shown in Fig 4A and 4B, the screw and plate stresses were markedly elevated during the excessive motion mode in both TACP and QACP implantation, as compared to normal motion mode. In particular, the stresses of the TACP screws and plates during the over extension motion were found to be the highest with values of 359.2 MPa and 97.2 MPa, respectively. The stresses of the QACP screws and the plates during the over extension motion were highest at values of 238.5 MPa and 86.7 MPa, respectively. Compared to the postoperative normal motion, the TACP screws and cervical plates had higher growth rates of average stress during excessive motion reaching values of 66.4% and 53.0%, respectively.

### Endplate stress

As presented in Fig 4C, compared to the postoperative normal motion, the stresses on the TACP and QACP C5-C6 endplates during excessive motion were remarkably high. In particular, the stresses of the TACP and QACP endplates during the over extension mode reached the highest value at 4.1 MPa and 7.5 MPa, respectively. Compared to the postoperative normal motion, the average increase rate of stress in the QACP C5-C6 endplates during excessive motion was 166.1%, which was much greater than 35.2% observed in the TACP system.

### Cage displacement

According to the cage relative displacement diagrams shown in Fig 4D, compared to the postoperative normal motion, peak displacements of the TACP and QACP cages during excessive motion remained high. In particular, the displacements of the TACP and QACP cages during over extension reached the largest value at 1 mm and 1.2 mm, respectively. Compared to the postoperative normal motion, the QACP cages had a higher average displacement increase rate at 45.2%, which was close to the 41.7% seen with the TACP system.

### Bone graft stress ratio

**Fig 5:**
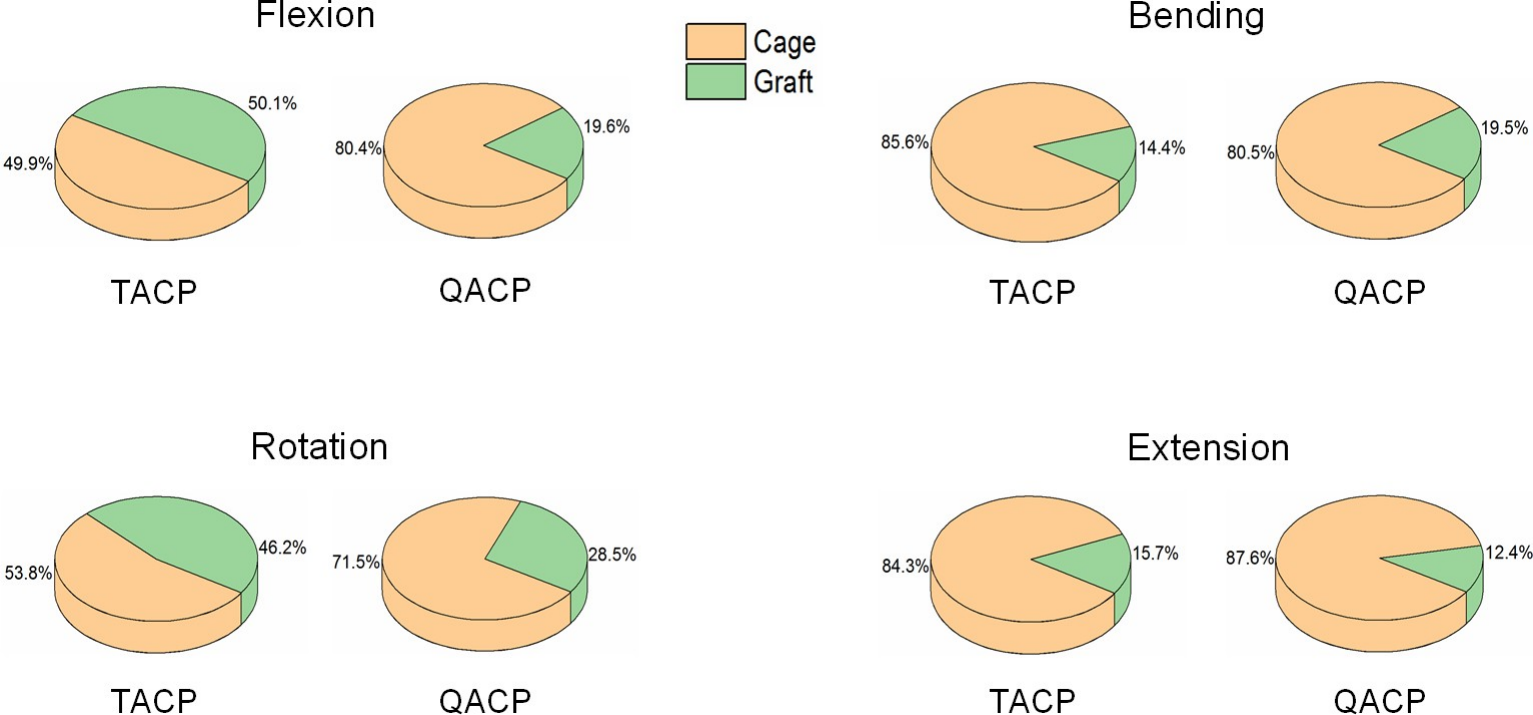
The proportion of stress between the fusion cage and the bone graft during normal exercise in the TACP and QACP implanted spines.

As shown in Fig 5, the TACP system had a higher bone graft ratio during flexion, rotation, and extension motions, which were 50.1%, 46.2%, and 15.7%, respectively. Alternately, the QACP system had a higher bone graft ratio during bending motion, reaching 19.5%. The average stress ratios of the TACP and QACP bone grafts during normal motion were 31.6% and 19.5%, respectively.

## Discussion

A typical single level ACDF surgery uses quadrilateral profile ACP to stabilize surgical segment. In this study, a triangular profile fusion system was designed, and evaluated the biomechanical performance difference between them, using FE analysis. According to our results, the stresses of the TACP and QACP screws were concentrated on the root and the TACP screws experienced higher stress peaks (Figs 3 and 4A). This is likely due to less number of screws, which under the same load, automatically increases the stress of a single screw, thereby raising the potential for TACP screw slippage and fracture.

Several studies have confirmed that endplates can properly support load on trabecular bone by uniformly distributing the load. Conversely, endplate removal can result in complications with strength and stiffness of the vertebral body, leading to abnormal load distribution and an increase in the risk of implant subsidence [25, 26]. As a result, it is clinically advisable to retain the endplates to prevent subsidence. Further studies have demonstrated that the magnitudes of stresses on the upper and lower endplates are directly related to cage subsidence. Hence, the endplate stress can be used as a performance measure for TACP and QACP implantation [27]. This study showed that during excessive motion states, the TACP screws and cervical plates had greater stress growing rates, while the endplate stress rate was relatively smaller. This suggests that TACP fares better under excessive motion as it can bear more load than QACP, thereby reducing cage subsidence risk.

Traditionally, the cage is stabilized *via* increased surface contact areas and friction forces, or by means of self-stretching, but there are reported cases of postoperative cage slippage. Although cage slipping is relatively rare, when it does occur it notifies failure of surgery and requires re-operation [28]. Therefore, cage slippage can also be used as an indicator of biomechanical performance of TACP and QACP implantation. As shown in Fig 4D, owing to the additional ACP system, both TACP and QACP performed well in terms of cage displacement. When challenged with excessive motion, however, TACP implantation experienced lower average slippage rate, suggesting this design to be superior in maintaining cage stability during excessive exercise.

Stress shielding occurs when bone density is lost due to the removal of stress to bone, often because of an implant. Stress shielding is also the main reason behind bone non-unions [29]. According to Wolff’s law, appropriate stress can stimulate bone growth [30]. Bone implantations often offer more rigidity to the cage as opposed to the bone graft, thereby forcing the cage to bear a larger load. With less load on the bone graft, the graft may fail to be fully stimulated and result in nonunion. Based on the stress ratios of the bone grafts and the cage in Fig 5, our TACP bone grafts showed higher stress ratios, during normal motion, thereby providing the best environment for bone fusion.

Our analysis also demonstrated that the cage displacement and the stresses of the screws, cervical plates, endplates appear the largest in both TACP and QACP implants during the over extension motion. Therefore, it is likely that a patient’s postoperative over extension motion is the cause of fusion failure. In ACP-related complications, Ning et al. [31] reported that the over extension of the neck was most responsible for screw fractures and cervical plate failures in 2233 patients treated with the ACP plates. During the extension motion, because of less ligament engagement in the cervical spine, and due to the thickness of the anterior part of intervertebral disc relative to the posterior, greater loads are introduced to the screws, cervical plates, and cages, as compared to other types of motions. Therefore, ACP implanted patients should be advised to avoid such postoperative extension motion.

There were some limitations in this study. Firstly, this study established a FE model based on the cervical CT data of one healthy volunteer. Therefore, the analyzed data of post implantation, may not be representative of the general population. But similar to other FE biomechanical analyses, FE method aimed to provide the trend comparison, rather than actual statistical data comparison. Secondly, the intact cervical spine model and the two implanted models did not consider the muscular structure, during the modeling process, and therefore, could not precisely simulate the motion of the cervical spine. This may have affected the FE analysis results. In addition, the angle of screw implantation, cage design, and other parameters selected in this study were derived from literature and assumptions. Hence, the conclusions from FE analysis were also based on the same assumptions. Therefore, caution must be taken when the above discussion is extrapolated to the overall biomechanics performance after TACP or QACP implantation. Finally, other factors, such as the angle and depth of screw implantation may have some effect on the performance of the implanted TACP or QACP. The biomechanics impact of implantation should be investigated further when these factors are coupled together.

## Conclusions

In summary, according to our study, the TACP system has multiple advantages over the QACP system during both normal and excessive motions. In terms of the biomechanical performance, TACP distributes the load more flexibly and reduces the risks of cage subsidence and slippage. TACP bone grafts also experience a higher stress ratio thereby stimulating bone growth and improving chances of fusion. There is, however, one disadvantage to using the TACP system. TACP screws have a greater potential of slippage and fracture due to fewer screws support a particular load. This disadvantage should be considered when constructing this design for a potential patient. Being an exploratory study, this work was not intended as a surgical guideline, but to provide new ideas and theoretical basis for the design of the anterior internal fixation system, so other researchers can conduct follow-up studies. Therefore, the actual effect of implantation still needs further clinical evaluation and in-depth investigation.

## Supporting information

S1 Table Comparative analysis data of two fixation systems during different neck motion patterns. (XLSX)
